# Phylogeny of *Salix* subgenus *Salix* s.l. (Salicaceae): delimitation, biogeography, and reticulate evolution

**DOI:** 10.1186/s12862-015-0311-7

**Published:** 2015-03-04

**Authors:** Jie Wu, Tommi Nyman, Dong-Chao Wang, George W Argus, Yong-Ping Yang, Jia-Hui Chen

**Affiliations:** Key Laboratory for Plant Biodiversity and Biogeography of East Asia, Kunming Institute of Botany, Chinese Academy of Sciences, Kunming, 650201 P. R. China; Plant Germplasm and Genomics Center, Kunming Institute of Botany, Chinese Academy of Sciences, Kunming, 650201 P. R. China; Institute of Tibetan Plateau Research at Kunming, Kunming Institute of Botany, Chinese Academy of Sciences, Kunming, 650201 P. R. China; Department of Biology, University of Eastern Finland, P.O. Box 111, FI-80101 Joensuu, Finland; Canadian Museum of Nature, Ottawa, ON Canada K2P 2R1

**Keywords:** *Salix* subgenus *Salix*, Phylogeny, Biogeography, Reticulate evolution, Character evolution

## Abstract

**Background:**

The taxonomy and systematics of *Salix* subgenus *Salix* s.l. is difficult. The reliability and evolutionary implications of two important morphological characters (number of stamens, and morphology of bud scales) used in subgeneric classification within *Salix* remain untested, and a disjunct Old–New World distribution pattern of a main clade of subgenus *Salix* s.l., revealed by a previous study, lacks a reasonable explanation. To study these questions, we conducted phylogenetic analyses based on 4,688 bp of sequence data from four plastid (*rbcL*, *trnD–T*, *matK*, and *atpB–rbcL*) and two nuclear markers (ETS and ITS) covering all subgenera of *Salix*, and all sections of subgenus *Salix* s.l.

**Results:**

Subgenus *Salix* came out as para- or polyphyletic in both nrDNA and plastid trees. The plastid phylogeny successfully resolved relationships among the major clades of *Salix*, but resolution within subgenus *Salix* s.l. remained low. Nevertheless, three monophyletic groups were identifiable in subgenus *Salix* s.l.: the ‘main clade’ of subgenus *Salix* s.l., with New and Old World species being reciprocally monophyletic; the section *Triandroides* clade; and the subgenus *Pleuradenia* clade. While nrDNA regions showed higher resolution within subgenus *Salix* s.l., they failed to resolve subgeneric relationships. Extensive, statistically significant gene-tree incongruence was detected across nrDNA–plastid as well as nrDNA ETS–ITS phylogenies, suggesting reticulate evolution or hybridization within the group. The results were supported by network analyses. Ancestral-state reconstructions indicated that multiple stamens and free bud scales represent the plesiomorphic states within *Salix*, and that several significant shifts in stamen number and bud scale morphology have occurred.

**Conclusions:**

Subgenus *Salix* s.l. is not monophyletic, and the evolutionary history of the subgenus has involved multiple reticulation events that may mainly be due to hybridization. The delimitation of subgenus *Salix* s.l. should be redefined by excluding section *Triandrae* and subgenus *Pleuradenia* from it. The evolutionary lability of bud-scale morphology and stamen number means that these characters are unreliable bases for classification. The disjunct Old–New World distribution of subgenus *Salix* s.l. appears to be linked to the profound climatic cooling during the Tertiary, which cut off gene exchange between New and Old World lineages.

**Electronic supplementary material:**

The online version of this article (doi:10.1186/s12862-015-0311-7) contains supplementary material, which is available to authorized users.

## Background

The genus *Salix* L. of Salicaceae, commonly known as willows, consists of some 450–520 species, is distributed mainly in the Northern Hemisphere, and is one of the main groups of trees and shrubs in the North Temperate Zone [1-3]. Because of their diversity, and because *Salix* species form an important resource for innumerable insect and mammalian herbivores [1,3], there is a clear need for understanding the evolutionary history of the group.

Unfortunately, the taxonomy and systematics of *Salix* have proven extremely difficult because of their dioecious reproduction, simple flowers, common natural hybridization, and large intraspecific phenotypic variation [1,3-5]. Reflecting these difficulties, *Salix* was once split into at least 35 genera (as reviewed by Argus [6]), but numerous molecular-phylogenetic studies have shown that *Salix* is a robust monophyletic group that encompasses all of the putative generic segregates [7-11]. This indicates that *Salix* is a natural group that should not be further split at the generic level, and so some recent systems e.g., [1,12] have opted to treat segregate genera, such as *Chosenia* and *Toisusu*, as members of *Salix*.

A widely used *Salix* classification system was proposed by Skvortsov in 1968 ([13]; revised and translated into English in 1999 [3]). In this system, he divided the Salicaceae into three genera, *Populus, Chosenia*, and *Salix*. Within *Salix*, Skvortsov recognized three subgenera: *Salix, Chamaetia*, and *Vetrix*. He also suggested that the subgenera *Urbaniana* and *Longifoliae* could be recognized, but did not do so. Because subgenus *Salix* as used by Skvortsov was later divided into several smaller subgenera, for the convenience of discussion, we will below refer to it as subgenus *Salix* s.l.

**Figure 1:**
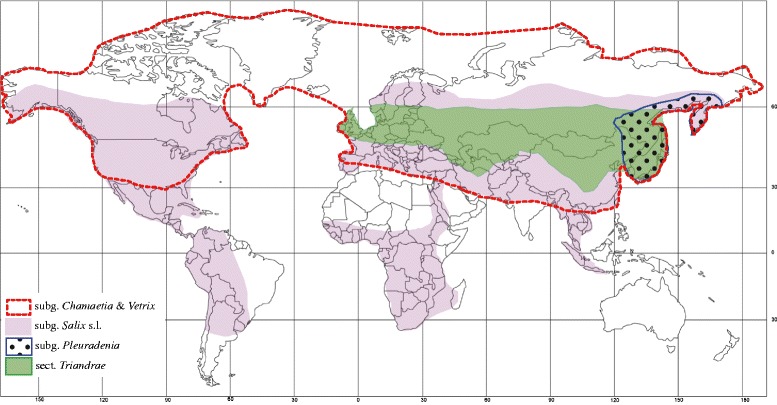
**Geographic distributions of the main lineages of** ***Salix*** **.**

Subgenus *Salix* s.l. is primarily distributed in the warm Temperate Zone and partially in tropical regions (Figure 1) [3]. The subgeneric and especially sectional division of subgenus *Salix* s.l. is in a chaos: section names and their delimitation in different systematic treatments differ, a main reason being that most systems are based on localized floras. Furthermore, different systems of subgenus *Salix* s.l. (as well as *Salix* on the subgeneric level) use bud-scale morphology (margin free or connate) and the number of stamens (two or multiple) as important criteria [1,3,4], but whether taxa classified according to these two traits are reasonable remains unknown.

After summarizing the main recent *Salix* systems [1-3,12], we found that subgenus *Salix* s.l. contains approximately 128 species. Most of them (109) are distributed in the Old World, and are placed into seven sections: *Tetraspermae*, *Urbanianae* (= subgenus *Pleuradenia*), *Wilsonia, Pentandrae*, *Triandrae*, *Octandrae*, and *Salix* (sometimes split into *Subalbae* and *Salix*). Only 19 species are found in the New World; these belong to the five sections *Longifoliae* (= subgenus *Longifoliae*)*, Humboldtianae*, *Floridanae*, *Maccallianae*, and *Salicaster*. However, while each section in this classification is found only in the New or Old World, morphological differences among species and sections are small. For example, the Old World sections *Tetraspermae* and *Pentandrae* appear to be closely related to the New World sections *Humboldtianae* and *Salicaster*, respectively, and Argus [1] treated section *Pentandrae* as a synonym of *Salicaster*, and section *Tetraspermae* as a synonym of *Humboldtianae*. Skvortsov’s sections *Humboldtianae* and *Pentandrae* likewise contain both New and Old World species.

Skvortsov [3] postulated that the subgenera *Chamaetia* and *Vetrix* are more closely related to each other than either is to subgenus *Salix*, and that subgenus *Salix* is a natural group, having the most in common with *Populus* and exhibiting “primitive” features in the structure of bracts, nectaries, androecium, and gynoecium. However, recent molecular-phylogenetic studies have cast doubt on the monophyly of subgenus *Salix* s.l. [8,9,11] and, based on morphological characters, the group has also been divided into at least five widely accepted subgenera: *Chosenia* and *Pleuradenia*, each containing only one species [12]; *Longifoliae*, with 8 species [1]; *Protitea*, with 33 species [1,12,14]; and *Salix*, with 85 species [1] (we refer to Argus’s [1] subgenus *Salix* as subgenus *Salix* s.str., in contrast to Skvortsov’s subgenus *Salix* s.l.). In Kimura’s [14] *Salix* system, subgenus *Chosenia* is a synonym of subgenus *Pleuradenia*.

The composition and further division of subgenus *Salix* s.l. is therefore a central issue in the systematics of *Salix*. However, sparse taxon sampling and use of low numbers of DNA markers in previous molecular-phylogenetic studies have led to restricted understanding of the classification and systematics within the group [9,15]. Our former study [9] also revealed a disjunct distribution pattern in the main clade of subgenus *Salix* s.l., so that Old and New Word species formed robust clades. By contrast, the subgenera *Chamaetia* and *Vetrix*, which also formed a robust monophyletic group, did not present similar trans-continental disjunctions in their (widely overlapping) geographic ranges (Figure 1). The low density of sampling of subgenus *Salix* s.l. representatives in the study unfortunately left the reliability of, and the reasons for, the disjunct distribution pattern unknown.

In this study, we examined representatives covering all major clades of *Salix* and all sections of subgenus *Salix* s.l. using six DNA regions: external transcribed spacer (ETS) and internal transcribed spacer (ITS) regions of nuclear ribosomal DNA (nrDNA), and partial sequences of the *rbcL* and *matK* genes, and *atpB–rbcL* and *trnD–T* non-coding spacers of plasmid chloroplast DNA. The main aims of our study were to: (1) establish a phylogeny of *Salix* subgenus *Salix* s.l. in order to evaluate the correctness and utility of present classification systems, (2) identify possible inconsistencies between nrDNA and plastid phylogenies, and to explore possible causes for it, (3) estimate the divergence times of the major clades of *Salix*, in order to explain their current distribution patterns, and (4) assess the value of the two most important characters used in classification and systematics of *Salix*, *i.e.*, bud scale morphology and stamen number.

## Results

### Sequence variation

The most variable of the six regions sequenced is ETS, with the highest average sequence divergence value and proportion of parsimony-informative characters, while the other nrDNA region, ITS, also shows high variation but much less than ETS (Table 1). Sequence variation in the plastid regions is lower than for nrDNA, and *matK* shows the least variation. The plastid *trnD–T* region possesses the most indels and highest indel diversity in *Salix*, while indels are absent from *matK* and *rbcL* (Table 1). Detailed alignment and sequence characters’ information for the regions sequenced, and tree statistics from the phylogenetic analyses, are listed in Table 1.

**Table 1:** **Characteristics of data matrix and tree statistics from the ML and MP analyses**

	**ETS**	**ITS**	***rbcL***	***trnD–T***	***atpB–rbcL***	***matK***	**Combined plastid**
No. of taxa	57	57	56	56	56	56	56
Aligned length	304	682	1119	1017	760	787	3683
GC%	63.0	64.5	43.6	32.3	28.3	33.5	35.2
No. (%) of variable characters	136 (44.7)	157 (23.0)	77 (6.9)	95 (9.3)	60 (7.9)	54 (6.9)	286 (7.8)
No. (%) of parsimony-informative characters	71 (23.4)	57 (8.4)	34 (3.0)	43 (4.2)	24 (3.2)	20 (2.5)	121 (3.3)
Average sequence divergence with/without outgroup	0.054/0.043	0.022/0.015	0.008/0.008	0.011/0.009	0.008/0.007	0.006/0.004	-
No. of indel sites/indel events in *Salix*	6/6	16/13	-	209/33	49/19	-	-
No. of haplotypes with indels in *Salix*	6	15	-	25	11	-	-
Indel diversity in *Salix*	0.388	1.345	-	6.898	1.111	-	-
No. of MP trees	8	3	10	8	10	8	5
Tree length (MP)	181	180	126	92	60	57	347
CI (MP)	0.762	0.850	0.837	0.815	0.883	0.982	0.827
RI (MP)	0.874	0.909	0.935	0.917	0.945	0.991	0.909
HI (MP)	0.238	0.150	0.163	0.185	0.117	0.018	0.173

### Phylogenetic trees and networks

The ILD test comparing the ETS and ITS datasets indicated significant incongruence (*P* = 0.002), and many of the incongruent clades were well supported (BS ≥ 70%). Significant incongruence likewise was detected between the nrDNA and plastid datasets (both *P* < 0.001), but not among the plastid regions (all *P* > 0.2). Therefore, phylogenetic analyses were performed for ETS, ITS, and combined plastid datasets, but not for combined nrDNA or combined nrDNA and plastid datasets.

**Figure 2:**
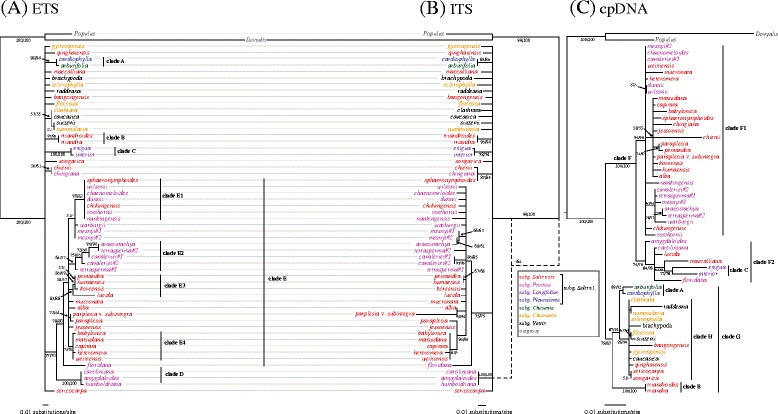
**Maximum likelihood trees based on (A) nrDNA ETS, (B) ITS, and (C) combined plastid sequence datasets.** ML/MP bootstrap support values above 50% are shown near branches. Dotted lines in **(B)** indicate branches that receive over 50% bootstrap support in the MP analysis, but which are not present in the ML topology. Bootstrap values of well-supported clades (BS ≥ 70%) are highlighted in bold.

While the topologies of nrDNA and plastid phylogenies are significantly incongruent, *Salix* was resolved as robustly monophyletic in all trees (Figure 2). The combined plastid tree (Figure 2C) successfully resolved relationships of major clades of *Salix*, and divided the genus into two well-supported clades (F and G) as well as many smaller subclades (A, B, C, F1, F2, and H). Subgenus *Salix* s.l. is not a monophylum in the plastid tree, although the majority of its species grouped into a large clade (F). Resolution inside the F clade is low, but Old and New World species are reciprocally monophyletic (subclades F1 and F2, respectively). Subgenus *Pleuradenia* and section *Triandrae* fell out this main F clade of subgenus *Salix* s.l.: two species of the latter (*S. triandra* and *S. triandroides*) formed a robust monophyletic group (clade B) as sister to a clade formed by subgenus *Pleuradenia* (clade A) and a robust monophyletic group (clade H) that includes all species of subgenera *Chamaetia* and *Vetrix* as well as three species of section *Salix* (*S. bangongensis, S. qinghaiensis*, and *S. sericocarpa*) and one species of section *Triandrae* (*S. songarica*). Among the subgenera consistently split from subgenus *Salix* s.l., *Pleuradenia* was resolved as monophyletic; subgenus *Longifoliae* was likewise monophyletic (clade C), but it was nested within the main *Salix* s.l. clade.

In contrast to the plastid tree, nrDNA ETS and ITS trees failed to resolve relationships of major clades of *Salix*, but they also indicate that subgenus *Salix* s.l. is not monophyletic (Figure 2A,B). Both of the nuclear trees exhibited a large basal polytomy, but especially the ETS tree shows higher resolution within subgenus *Salix* s.l. A large clade consisting of some 27 or 28 Old World subgenus *Salix* s.l. species (*S. sphaeronymphoides* falls outside this clade in the ITS tree) and two New World species (*S. lucida* and *S. floridana*) was present in the nrDNA trees (clade E in Figure 2). Several well-supported subclades of clade E were recognized (E1–E4), but most of them are incongruent, except for clade E4, which consists of species of section *Salix* (Figure 2). Species of section *Humboldtianae* form a monophyletic, well-supported sister group (clade D) of clade E in the ETS tree. In the ITS tree, section *Humboldtianae* is sister to the remaining *Salix* taxa, but this relationship is poorly supported (MP BS = 64%) and present only in the MP tree (Figure 2B).

**Figure 3:**
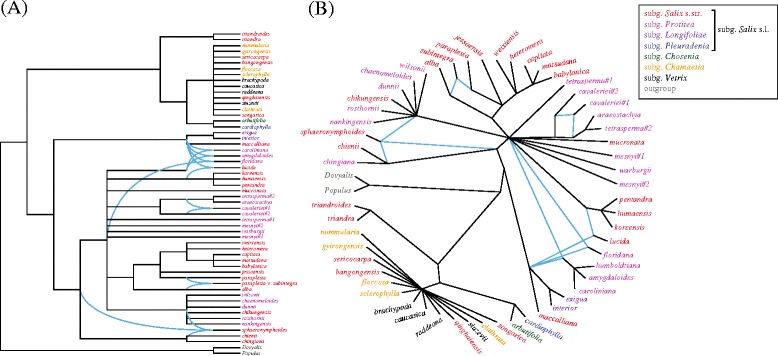
**Phylogenetic network of nrDNA ETS, ITS, and combined plastid ML trees with inferred reticulation events, drawn as (A) rectangular and (B) radial cladograms.**

Numerous (at least seven) reticulation events were detected in the network analysis (Figure 3). Notably, all of them are associated with taxa belonging to clade F of subgenus *Salix* s.l. in the plastid tree, whereas no reticulation events were detected in clade G (Figure 2C).

### Divergence times of the main clades of *Salix*

**Figure 4:**
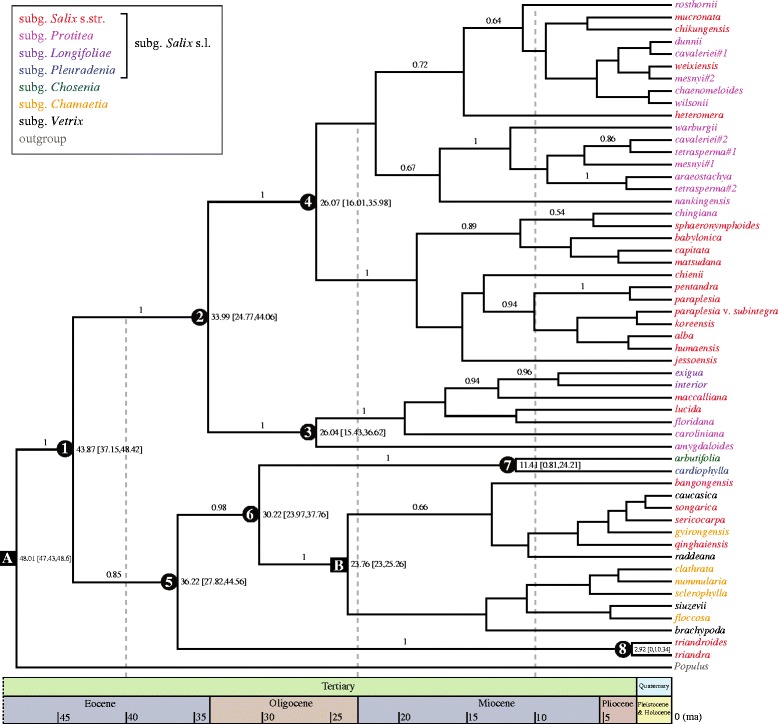
**Relaxed molecular-clock chronogram based on combined plastid sequence data.** Nodes used for calibration **(A** **and B)** are marked with black squares (see text for details). The main clades of *Salix* are numbered (1–8), and their estimated ages (with 95% HPD intervals in square brackets) are shown to the right of each node.

The crown-group age of *Salix* (node 1 in Figure 4) was estimated to be 43.87 Ma (95% HPD: 37.15–48.42 Ma), and the divergence time of the main clade of New and Old World subgenus *Salix* s.l. (node 2) was estimated at 33.99 Ma (95% HPD: 24.77–44.06 Ma). The divergence times of other main clades of *Salix*, estimated based on combined plastid datasets, are shown in Figure 4.

### Character evolution

**Figure 5:**
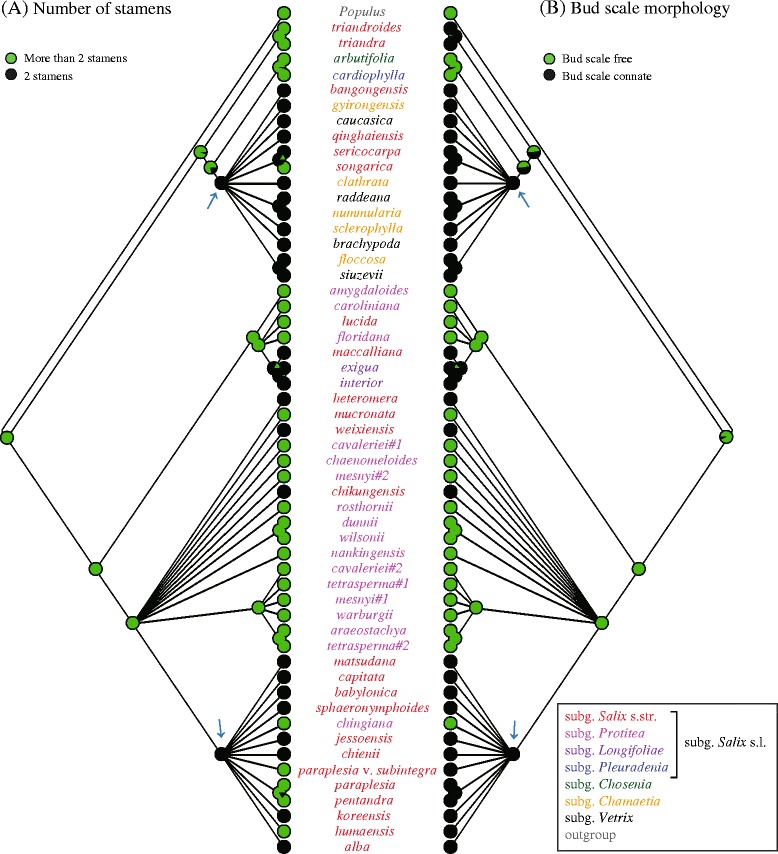
**Ancestral state reconstruction of (B) bud scale morphology and (A) number of stamens, according to maximum likelihood optimization of states across the ML tree based on combined plastid datasets.** Arrows indicate nodes with major character state shifts.

Our ancestral-state reconstructions show that multiple stamens and free bud scales represent the plesiomorphic states within both the genus *Salix* and the main clade of subgenus *Salix* s.l., while a shift to two stamens and connate bud scales occurred at the base of the lineage leading to *Chamaetia* and *Vetrix* (Figure 5). However, groups defined by the alternative states of neither character are monophyletic, because the number of stamens has repeatedly been reduced from multiple to two, and connate bud scales have originated multiple times convergently. Also reversals to the plesiomorphic condition in both characters can be identified across the tree.

## Discussion

### Taxonomic and systematic implications

The monophyly of the genus *Salix* in our results is consistent with inferences from previous phylogenetic analyses of morphological and molecular data e.g., [5,7,9,16,17], and support the view that *Salix* is a natural group that should not be further split at the generic level.

The species of subgenera *Chamaetia* and *Vetrix* fall into a robust monophyletic group in our plastid gene trees, but resolution within the clade is low, and the subgenera are not reciprocally monophyletic. This result is consistent with previous research [8,9,16], and supports the merging of *Chamaetia* with *Vetrix* [18]. Nevertheless, although it is clear that subgenus *Chamaetia* is morphologically closer to subgenus *Vetrix* than to subgenus *Salix*, it may be taxonomically useful to treat them as separate subgenera until more representatives of these two subgenera have been included in molecular studies.

In the subgenera divided from subgenus *Salix* s.l., *Pleuradenia* was established by Kimura in 1988 and consisted of *S. arbutifolia* and *S. cardiophylla* [14], to which he gave generic rank (*Chosenia* and *Toisusu*, respectively) in 1928 [19]. Ohashi [12] recognized them as two subgenera (*Chosenia* and *Pleuradenia,* respectively). In our results, *S. arbutifolia* and *S. cardiophylla* are consistently placed as sister species, which is consistent with former studies [9,15,20] and their morphological affinities (both possess pendulous catkins, have connate stamens with bracts at their base, and have deciduous stigmas after flowering), and supports the grouping of both species in subgenus *Pleuradenia* Kimura.

The American subgenus *Longifoliae* was established by Argus [21] based on molecular, anatomical, developmental, chemical, genetic, and morphological evidence. Our results indicate that *Longifoliae* constitutes an independent lineage of *Salix*, but since it is nested inside the subgenus *Salix* s.l. clade in our plastid trees, its subgeneric rank needs further confirmation in the future. The subgenera *Salix* s.str. and *Protitea* are clearly poly- or paraphyletic in our results. Although some subclades were recognized for both of these subgenera, the subclades seem not to follow any distribution pattern nor share any obvious morphological traits, and no sections were recognized as monophyletic.

Previous studies have indicated that *S. triandra* is in some traits somewhat unique as compared to other *Salix* species, and that it might therefore constitute a distinct subgenus [9,22]. However, these investigations both included *S. triandra* as the sole representative of section *Triandrae*. Our analysis, which included all three species of section *Triandrae*, reveal that the group, as currently defined [12], is not monophyletic, because *S. songarica* falls outside the *S. triandra–S. triandroides* clade (clade B in Figure 2). Section *Triandrae* is defined mainly by the presence of 3 stamens, but this character differs among and within species. Most flowers of section *Triandrae* have 3 stamens, but *S. triandra* occasionally has 2, 4, or 5 stamens, while *S. songarica* sometimes possesses 4 stamens [2], indicating that this section cannot be reliably defined based on stamen number. We suggest treating section *Triandrae* as a subgenus, but delimited so that it includes only *S. triandra* and *S. triandroides*.

Four species of *Salix* s.l. (*i.e.*, *S. qinghaiensis*, *S. sericocarpa*, *S. bangongensis*, and the aforementioned *S. songarica*) are grouped with the subgenera *Chamaetia* and *Vetrix* in the plastid tree; in the nrDNA tree, these species are placed in a polytomy outside the large subgenus *Salix* s.l. clade. The network analysis (Figure 3) indicates no reticulations involving these species, suggesting that their positions are congruent in all phylogenetic trees, and that they have a closer relationship with subgenera *Chamaetia* and *Vetrix* than with subgenus *Salix* s.l. All of these species have two stamens, except for *S. songarica*, which has three (a reversion as indicated by our character-evolution analysis, Figure 5). This indicates that these four species should be treated as members of subgenera *Chamaetia* or *Vetrix.*

### Phylogenetic conflict

Phylogenetic incongruence between different sequence datasets has been demonstrated in many plant groups [23,24]. As summarized by Zou and Ge [24], stochastic (sampling) errors, systematic errors (long-branch attraction, *i.e.*, long branches tend to group together in MP analyses even if they are distantly related [25]), and biological factors could give rise to gene-tree conflicts.

Methods usually used to diminish the first two conflicts include increasing the number of characters and using an appropriate phylogenetic analysis method [24]. We employed six DNA markers to increase character number, and analyzed the data using both MP and ML, so stochastic and systematic errors can both be excluded as reasons for the gene-tree conflicts observed. Instead, the incongruent phylogenies may be caused mainly by biological factors, including horizontal gene transfer (HGT), undetected paralogs, incomplete lineage sorting (ILS), and hybridization/introgression [23,26]. Chloroplasts seem essentially immune to HGT (as reviewed by Richardson and Palmer [27]), meaning that HGT is not a likely cause for the gene-tree discordance. Ribosomal genes exhibit high copy numbers [28], and concerted evolution, which tends to homogenize sequences of rDNA arrays, may be incomplete [28,29]. ITS and ETS sequences likewise have relatively high levels of homoplasy [28]. This is exactly the case in our results (Table 1), so it is possible that ortholog/paralog confusion and a higher level of homoplasy caused part of the detected gene-tree conflicts; further studies are needed to clarify these issues. However, because lineage sorting of ancestral genotypes is a stochastic process, it is not expected to follow any geographic pattern [30]. By contrast, some of the clades in our trees clearly reflect geographic patterns (e.g., Old and New World species of the main clade of subgenus *Salix* s.l. form reciprocally monophyletic groups in the plastid tree). Therefore, unlike Hardig et al. [10], we think ILS is most likely insufficient to explain the gene-tree incongruence.

Morphological and molecular studies have shown that hybridization among *Salix* species is an important source of variability [1,3,4], and that hybrids are frequently able to backcross and to introgress [31-33]. Our network analysis indicated that reticulate evolution is common in subgenus *Salix* s.l., suggesting that hybridization/introgression is the main reason for the extensive gene-tree conflicts. For example, Clade E in the nrDNA trees (Figure 2A,B) consists of subgenus *Salix* s.l. species; all but two (*S. floridana* and *S. lucida*) are Old World species, with *S. floridana* being sister to the remaining species of this clade. However, the placement of these two New World species conflicts with the plastid tree, in which they are nested in the New World clade within subgenus *Salix* s.l. Because there was no gene flow between the ancestors of the New and Old World lineages of subgenus *Salix* s.l. (see Discussion below), this may be primarily due to ancient hybridization as indicated by the network analysis (Figure 3). This is consistent with morphological traits: *S. floridana* resembles the Old World subgenus *Salix* s.l. species *S. tetrasperma* and *S. rosthornii*, and it is assumed to be a relict or a descendant of the Arcto–Tertiary flora [34]. Furthermore, *S. lucida* is similar to the Old World subgenus *Salix* s.l. species *S. pentandra* in leaf and catkin morphology [34]. Likewise, the other conflicting clades can also explained by hybridization; these species include *S. caroliniana*, *S. amygdaloides*, *S. cavaleriei*, *S. paraplesia* var. *subintegra*, and *S. sphaeronymphoides.*

### Biogeographic implications

The most striking distribution pattern of *Salix* revealed by our molecular- phylogenetic study is the New–Old World disjunction within the main clade of subgenus *Salix* s.l. in the plastid tree. Similar disjunctions are not found within the subgenera *Chamaetia* and *Vetrix*, despite the fact that they are also distributed widely in across the Northern Hemisphere. The divergence times of the main clade of New and Old World subgenus *Salix* s.l. and subgenera *Chamaetia* and *Vetrix* were estimated at 33.99 (about early Oligocene) and 23.76 mya (about lower Oligocene), respectively. During the middle of the upper Oligocene, the physical continuity of the Bering Land Bridge (BLB) is certain, but questioned for the North Atlantic Bridge (NALB) [35]. However, while paratropical groups presumably could exchange rather freely across the BLB during the early Paleocene and Eocene, intercontinental dispersal of such thermophilic elements was increasingly restricted by subsequent climatic cooling [35,36]. Extant representatives of subgenus *Salix* s.l. mostly inhabit sub-tropical and partially tropical regions (Figure 1), and it is therefore reasonable to assume that the subgenus was ancestrally adapted to temperate conditions, and that they could disperse freely between the New and Old Worlds via the BLB only during their early history. On the other hand, willow seeds have fine hairs and can travel hundreds of kilometers by air, and *Salix* species in the Svalbard archipelago have colonized the islands by repeated long-distance dispersal from Scandinavia [37]. Hence, the possibility of dispersal between the New and Old Worlds by island-hopping via the NALB during the Eocene and Oligocene boundary cannot be ruled out. However, regardless of the exact route taken, subsequent climatic cooling in the Tertiary cut off the floral exchange between New and Old World lineages of subgenus *Salix* s.l., resulting in the intercontinental disjunction pattern seen in our phylogenetic trees.

By contrast, most species of the subgenus *Chamaetia* and some species of *Vetrix* are Arctic–Alpine taxa that are well adapted to cold, hostile environments. Floristic migrations over the BLB were primarily controlled by climate in most of the Cenozoic [38,39], and large parts of the Arctic and Subarctic in northwest America and eastern Siberia (*i.e.*, Beringia) were never glaciated [38,40]. These factors indicate that the climatic cooling of the late Tertiary [36] facilitated intercontinental gene exchange within the cold-adapted subgenera *Chamaetia* and *Vetrix*, and also resulted in widely Holarctic distributions in many extant species within subgenus *Chamaetia*.

The *S. cardiophylla*–*S. arbutifolia* (*i.e.*, subgenus *Pleuradenia* Kimura) clade has a limited distribution area in northeastern Asia (Figure 1). The crown age of it was estimated at 11.41 mya (95% HPD: 0.81–24.21 mya). The common ancestors of this group most likely diverged in northeastern Asia, and when and after they diverged, there were factors limiting north-to-south floristic exchange in central Asia (temperature and moisture) and east-to-west exchange across central China (moisture) [35]. Therefore, the ancestors of this clade could not disperse to Eurasia and the New World.

### Character evolution

The number of stamens has been thought to be a key trait reflecting the evolutionary history of *Salix*, and it is still one of the main characters used in the classification of *Salix* at the subgeneric level [1,3,12,41,42]. Stamen number within *Salix* varies from 2 to 12, with filaments being either free or partly to completely connate [2], but only 49 *Salix* species (~10%) have multiple stamens [1]. Our results revealed a trend of reduction in stamen number from multiple to two in several different lineages of *Salix*. As a result, stamen number states are paraphyletic across the phylogenetic tree, and the frequent changes indicate that the number of stamens constitutes an unreliable basis for *Salix* classification.

Interestingly, it seems that stamen number is related to species diversity in different *Salix* lineages, because the subgenera *Chamaetia* and *Vetrix*, which together account for ~73% of the species within the genus, all have two stamens. Pollination in *Salix* is almost exclusively performed by insects [3], and insect pollination predominates also in the few species that are ambophilous [43]. Because male flowers reward biotic pollinators with both pollen and nectar, while females only produce nectar, some biotic pollinators might discriminate against female flowers, resulting in reduced female fertility and favoring the evolution of wind pollination or pollination by less-discriminating pollinators [44,45]. Pollinator discrimination can also be avoided by female flowers by producing more nectar than male flowers [46-48]. Here we speculate that the reduction of stamen number might be an alternative adaptation, in that it may reduce pollen production and therefore might be favored by natural selection. Reductions in the number of stamens may also allow more precise pollen transfer by specialist insect pollinators and, consequently, lead to less expense of pollen and nectar [49-51]. This type of selective advantage of specialist pollination may have played an important role in the diversification of *Salix*, and could partly result in unequal species diversity in different *Salix* lineages. Further efforts are needed to test this hypothesis.

Bud scale morphology is another key characteristic used in traditional subgenus-level classifications of *Salix*. About 91% of *Salix* species (including all species of subgenera *Chamaetia* and *Vetrix*) have connate bud scales. Our ancestral-state reconstruction showed that bud scale margins changed from free to connate independently in different lineages. Our divergence-time estimates indicate that *Salix* originated approximately in the middle of the upper Oligocene. As mentioned above, the Tertiary was a time of cooling climates [52], and connate bud scales could protect the apical meristem more effectively than free ones; therefore, the presence of connate bud scales could have been selectively favored in cold-adapted lineages such as the subgenera *Chamaetia* and *Vetrix*.

## Conclusions

Our nrDNA and plastid trees revealed that, while the genus *Salix* constitutes a robust monophyletic group, this is not the case for subgenus *Salix* s.l. Among the subgenera previously split from subgenus *Salix* s.l., only *Longifoliae* and *Pleuradenia* are supported as being monophyletic. The delimitation of subgenus *Salix* s.l. should be redefined so that sections *Triandrae* and *Urbanianae* (= subgenus *Pleuradenia*), as well as a few additional species (*S. bangongensis*, *S. qinghaiensis*, *S. sericocarpa*, and *S. songarica*), are excluded from it. Our phylogeny-based ancestral-state reconstructions indicated that the presence of multiple stamens and free bud scales are plesiomorphic within *Salix*, but also that shifts from multiple to two stamens and from free to connate bud-scale margins have occurred repeatedly; this means that these traditionally used morphological traits constitute an unreliable basis for subgenus-level classifications.

Extensive gene-tree conflicts between nrDNA and plastid phylogenies as well as between nrDNA ETS and ITS regions primarily appear to be due to hybridization and reticulate evolution. This interpretation is in line with the recent results of Percy et al. [5], who found widespread sharing of plastid haplotypes and even signs of possible trans-specific selective sweeps within *Salix*. In the plastid tree, Old and New World representatives of the main clade of subgenus *Salix* s.l. are reciprocally monophyletic; our biogeographic analysis based on a fossil-calibrated phylogenetic tree indicates that the disjunction results from climatic cooling during the late Tertiary, which cut off northern dispersal routes and, hence, genetic exchange between the continents. By contrast, in the more cold-tolerant subgenera *Chamaetia* and *Vetrix*, continuous floristic exchange via the BLB and/or NALB apparently prevented the formation of similar disjunctions. In the future, detailed genus-level phylogenetic analyses of *Salix* could provide important insights into the historical roles of the BLB and NALB as climatic filters for dispersal in lineages with different thermal tolerances (cf. [36]).

## Methods

### Plant material, DNA extraction, and sequencing

We sampled 55 specimens representing 51 *Salix* species (see Additional file 1: Table S1). The taxon sample includes nine species from the subgenera *Chamaetia* and *Vetrix*, and 47 specimens representing 41 species of subgenus *Salix* s.l., covering all of the subgenera and sections that have been recognized. *Populus* and *Dovyalis* were used as outgroups.

DNA was extracted from silica-gel dried leaves or from herbarium specimens using the CTAB method of Saghai-Maroof et al. [53] as modified by Doyle & Doyle [54]. PCR was performed in 50 μl volumes with the following reaction components: 1 μl template DNA (50 ng/μl), 33.75 μl H_2_O, 5 μl 10X PCR buffer (Mg^2+^ free), 4 μl MgCl_2_ (25 mM), 4 μl dNTP mix (2.5 mM of each nucleotide), 0.25 μl TakaRa Taq (5 U/μl), and 1 μl of each primer (20 μM). The following primers were used for both amplification and sequencing of the six employed markers: “18 s-IGS” and “Bur-ETS1F” for ETS [55,56], “ITS-a” and “ITS-d” for ITS [11], “trnD^GUC^F” and “trnT^GGU^” for *trnD–T* [57], “atpB-1” and “rbcL-1” for *atpB–rbcL* [58], “1 F” and “1024R” for *rbcL* [59], and “3F_KIM f” and “1R_KIM r” for *matK* [60]. PCR products were purified with a multifunction DNA Purification Kit (BioTeke Inc.), and then sequenced using an ABI Prism BigDye Terminator v3.1 Cycle Sequencing Kit (Perkin-Elmer Applied Biosystems) and an ABI Prism 377 Automated DNA sequencer (Perkin-Elmer Applied Biosystems).

### Phylogenetic analyses

Sequences were assembled using Geneious v. 5.4 [61] and aligned with Muscle [62]. After manual correction in Geneious, regions with ambiguous alignments were excluded from the analysis. Average sequence divergences (*i.e.*, pairwise distance) were estimated with Kimura’s [63] two-parameter method in Mega v. 5.2 [64]. Numbers of indel sites, events, and diversity indices were calculated in DnaSP v. 5 [65].

Phylogenetic analyses were performed based on maximum-parsimony (MP) and maximum-likelihood (ML) criteria. MP analyses were performed with PAUP* v. 4.0b10 [66]. Gaps were treated as missing data, and characters were assumed to be unordered. Optimal trees were found using a heuristic search with the following options: tree-bisection-reconnection (TBR) branch-swapping, MulTrees option in effect, starting tree obtained via stepwise addition, trees held at each step = 5, and MaxTrees = 100. Branch support was estimated using bootstrapping (BS) with 2000 replicates [67]. The ML analysis employing a GTR + Γ + I model of substitution for all datasets was run in RAxML v. 7.2.8 [68], and a bootstrap analysis of 2000 replicates was performed simultaneously (option “-f a”).

### Tests for data incongruence and evaluation of opposing hypotheses

Topological incongruence among partitions was tested using the incongruence length difference (ILD) test [69] in PAUP, with uninformative characters excluded and 500 replicates. Strong incongruence is defined as ILD values resulting in a *P* < 0.01 [70]. Because the efficacy of the ILD test has been questioned [71-73], we also compared phylogenetic trees reconstructed from individual data sets. Well-supported (BS ≥ 70%, as indicated by Hillis and Bull [74]) and conflicting clades are defined as incongruent [75]. Combined analyses were not conducted when both approaches indicated significant incongruence among the datasets.

### Phylogenetic network analysis

Incongruence between the nrDNA ETS and ITS as well as combined plastid datasets were further explored using a phylogenetic network approach [76,77]. ML trees of nrDNA ETS, ITS, and combined plastid datasets were used to infer phylogenetic networks, and weakly supported branches (BS < 70%) were collapsed into multifurcations. A Network consensus tree was then constructed under the galled network consensus algorithm implemented in Dendroscope v. 3.2.8 [76].

### Divergence time estimation

To estimate divergence times among lineages, we employed the combined plastid *rbcL, matK, atpB–rbcL*, and *trnD–T* dataset, which successfully resolved relationships of major clades of *Salix*. Because a likelihood ratio test [25] rejected the assumption of rate constancy (= clock-like evolution) across the tree, we instead used the Bayesian uncorrelated log-normal relaxed molecular clock approach [41] as implemented in BEAST v. 1.5.4 [78] to estimate times of divergence and associated confidence intervals. The analysis was performed using a GTR + Γ + I substitution model with four rate categories and a Yule model prior on speciation. Posterior distributions of parameters were estimated using two independent MCMC analyses of 30,000,000 generations with a 10% burn-in. Samples from the two runs, which yielded similar results, were combined after checking convergence of the chains using Tracer v. 1.6 [79].

The Salicaceae has a rich fossil record, but reliably identifiable fossils with both catkins and leaves are rare [80]. An approximately 48-million-year-old fossil from the early Eocene in North America with well-preserved foliage and fruits was named *Populus tidwellii*, but its placement remains suspect because it possesses *Populus*-like infructescences but *Salix*-like leaves [81]. The immature fruits suggest that pseudoracemes may have characterized stem lineages within both *Populus* and *Salix*, and the lack of true racemes may indicate that early divergent members of the stem lineage of the *Populus* and *Salix* clades of Salicaceae s.l. were characterized by *Populus*-type capsules [81,82]. Most likely, *Populus tidwellii* represents the stem lineage leading to *Populus* and *Salix* [81]. *Pseudosalix*, another extinct, early divergent member of the stem lineage of the *Populus* plus *Salix* clade of Salicaceae s.l., was present at almost the same time [82]. Although *Salix* is rich in the fossil record [80] and most likely can be traced back to the early Eocene, most fossils are represented only by leaves, so it is possible that some of the *Salix*-like leaves represent extinct taxa such as *Pseudosalix* or other genera [82]. The earliest reliable *Salix* fossils with both catkins and leaves originate from the late Oligocene (about 23 million years ago) in Alaska and belong to subgenus *Vetrix* [80,83]. Based on the above, we used two fossil calibrations to place priors on the ages of nodes within the tree: the split between *Salix* and *Populus* (*i.e.*, the root node of Salicaceae s.str.) was assigned a normally distributed prior with a mean of 48 Ma and a standard deviation of 0.3, and the divergence between the subgenera *Chamaetia* and *Vetrix* was assigned an exponential distribution prior with a mean of 1 and offset (hard bound constraint) of 23 Ma. The exponential distribution was employed here because it has a long tail of diminishing probability towards older ages [84].

### Character evolution analyses

We reconstructed the evolutionary history of bud-scale morphology and stamen number by tracing the characters onto the ML tree of the combined plastid dataset (which successfully resolved relationships of major clades of *Salix*). Bud scale was scored as 0 (= margin free) or 1 (= margin connate), and stamen number as 0 (= more than two) or 1 (= two), and ancestral states were estimated using Mk1-model maximum-likelihood optimization in Mesquite v. 2.75 [85].

## Availability of supporting data

The data sets (all sequence alignments) supporting the results of this article are available in the Dryad repository [86], [http://dx.doi.org/10.5061/dryad.qr2vv]. All sequence data is available on Genbank (http://www.ncbi.nlm.nih.gov/genbank).

## Additional file

Additional file 1: Table S1. List of studied taxa, and voucher information for samples.
