# Supplementary material for: Phylogeny of Salix subgenus Salix s.l. (Salicaceae): delimitation, biogeography, and reticulate evolution
Source: BMC Evol Biol. 2015 Mar 4;15:31. doi: 10.1186/s12862-015-0311-7 (PMC4357182; doi:10.1186/s12862-015-0311-7)
Supplement: Additional file 1: Table S1. — List of studied taxa, and voucher information for samples. [file 12862_2015_311_MOESM1_ESM.docx]

**Table S1** List of studied taxa, and voucher information for samples

| **Taxon**^a^ | | **Voucher; locality; herbarium**^d^ | **GenBank accession number**^c^ | | | | | |
| --- | --- | --- | --- | --- | --- | --- | --- | --- |
|  |  |  | ITS | ETS | *rbcL* | *trnD-T* | *atpB-rbcL* | *matK* |
| **outgroup** | *Dovyalis hebecarpa* (Gardner) Warb. | Chen JH 790; Yunnan, China; KUN | KC415490 | KC415433 | KC415321 | FJ788595 | FJ788540 | KC415377 |
|  | *Populus cathayana* Rehder | Chen JH 139; Jilin, China; KUN | KC415491 | KC415434 | KC415322 | FJ788596 | FJ788541 | KC415378 |
| ***Salix*** |  |  |  |  |  |  |  |  |
| **subg. *Choseniae***  (Nakai) H. Ohashi | *Salix arbutifolia* Pall. | Chen JH 134; Jilin, China; KUN | KC415495 | KC415438 | KC415326 | FJ788620 | FJ788535 | KC415382 |
| **subg. *Longifoliae***  (Andersson) Argus | *S. exigua* Nutt.^b^ | Doug G 2344; Oregon, USA; A | KC415510 | KC415453 | KC415341 | FJ788627 | FJ788563 | KC415397 |
|  | *S. interior* Rowlee^b^ | s.n.; Minnesota USA; A | KC415517 | KC415460 | KC415347 | KC415299 | KC415517 | KC415403 |
| **subg. *Pleuradenia*** Kimura |  |  |  |  |  |  |  |  |
| sect. *Urbanianae* C.K. Schneid. | *S. cardiophylla* Trautv. & Mey. | Tanaka T s.n.; Honshu, Japan; KUN, CANM | KC415500 | KC415443 | KC415331 | FJ788619 | FJ788556 | KC415387 |
| **subg. *Protitea*** Kimura |  |  |  |  |  |  |  |  |
| sect. *Floridanae* Dorn | *S. floridana* Chapm.^b^ | Iohn RM 13967; USA; KUN | KC415512 | KC415455 | KC415343 | FJ788629 | FJ788529 | \| KC415399 \| \| --- \| |
| sect. *Humboldtianae* Kimura | *S. amygdaloides* Andersson | Argus GW 14299; Ontario, Canada; CANM | KC415493 | KC415436 | KC415324 | KC415285 | KC415548 | KC415380 |
|  | *S. humboldtiana*^b^ | Ritter N et al. 2135; Mizque, Bolivia; A | KC415516 | KC415459 | - | - | - | - |
|  | *S. caroliniana* Michaux^b^ | Arnold T 12149; Virginia, USA; A | KC415520 | KC415463 | KC415350 | KC415302 | KC415565 | KC415406 |
| sect. *Tetraspermae* Andersson | *S. araeostachya* C.K. Schneid. | Wang DC 687; Guangxi, China; KUN, CANM | KC415494 | KC415437 | KC415325 | KC415286 | KC415549 | KC415381 |
|  | *S. tetrasperma* Roxb. #1 | Chen JH 547; Tengchong, Yunnan, China; KUN, CANM | KC415540 | KC415483 | KC415370 | KC415315 | KC415577 | KC415426 |
|  | *S. tetrasperma* Roxb. #2 | Chen JH 490; Tonghai, Yunnan, China; KUN, CANM | KC415541 | KC415484 | KC415371 | KC415316 | KC415578 | KC415427 |
| sect. *Wilsonia* K.S. Hao ex C.F. Fang & A.K. Skvortsov | *S. cavaleriei* H. Léveil. #1 | Chen JH 550; Yunnan, China; KUN | KC415502 | KC415445 | KC415333 | KC415290 | KC415553 | KC415389 |
|  | *S. cavaleriei* H. Léveil. #2 | Chen JH 1038; Yunnan, China; KUN | KC415503 | KC415446 | KC415334 | KC415291 | KC415554 | KC415390 |
|  | *S. chaenomeloides* Kimura | HB 265; Nagoya, Japan; KUN | KC415504 | KC415447 | KC415335 | KC415292 | KC415555 | KC415391 |
|  | *S. chingiana* K.S. Hao ex C.F. Fang & A.K. Skvortsov | Chen JH 458; Qinghai, China; KUN, CANM | KC415507 | KC415450 | KC415338 | KC415295 | KC415558 | KC415394 |
|  | *S. dunnii* C.K. Schneid. | Chen JH 362; Jiangxi, China; KUN, CANM | KC415509 | KC415452 | KC415340 | KC415296 | KC415559 | KC415396 |
|  | *S. mesnyi* Hance #1 | Wang DC 663; Zhejiang, China; KUN, CANM | KC415524 | KC415467 | KC415354 | KC415304 | KC415567 | KC415410 |
|  | *S. mesnyi* Hance #2 | Wang DC 676; Hunan, China; KUN, CANM | KC415525 | KC415468 | KC415355 | KC415305 | KC415568 | KC415411 |
|  | *S. nankingensis* C. Wang & S.L. Tung | Chen JH 347; Jiangsu, China; KUN, CANM | KC415527 | KC415470 | KC415357 | KC415307 | KC415570 | KC415413 |
|  | *S. rosthornii* Seemen | Chen JH 341; Zhejiang, China; KUN, CANM | KC415533 | KC415476 | KC415363 | KC415310 | KC415573 | KC415419 |
|  | *S. warburgii* Seemen | Chao s.n.; Taiwan, China; KUN | KC415544 | KC415487 | KC415374 | KC415318 | KC415580 | KC415430 |
|  | *S. wilsonii* Seemen ex Diels | Chen JH 345; Jiangsu, China; KUN, CANM | KC415546 | KC415489 | KC415376 | KC415320 | KC415582 | KC415432 |
| **subg. *Salix*** |  |  |  |  |  |  |  |  |
| sect. *Triandrae* Dumort. | *S. songarica* Andersson | Wang LL s.n.; Xinjiang, China; KUN, CANM | KC415538 | KC415481 | KC415368 | KC415313 | KC415575 | KC415424 |
|  | *S. triandra* L.^c^ | Chen 136; Heilongjiang, China; KUN | KC415542 | KC415485 | KC415372 | FJ788621 | FJ788560 | KC415428 |
|  | *S. triandroides* W.P. Fang | Chen JH 558; Hunan, China; KUN, CANM | KC415543 | KC415486 | KC415373 | KC415317 | KC415579 | KC415429 |
| sect. *Maccallianae* Argus | *S. maccallianae* Rowlee^b^ | Welby RS 27730; USA; A | KC415522 | KC415465 | KC415352 | FJ788628 | FJ788542 | KC415408 |
| sect. *Octandrae* Andersson | *S. mucronata* Thunb. | s.n.; Nagoya, Japan; KUN | KC415526 | KC415469 | KC415356 | KC415306 | KC415569 | KC415412 |
| sect. *Pentandrae* C.K. Schneid. | *S. humaensis* Y.L. Chou & R.C. Chou | Wang DC 485; Heilongjiang, China; KUN | KC415515 | KC415458 | KC415346 | KC415298 | KC415561 | KC415402 |
|  | *S. paraplesia* C.K. Schneid. | Boufford DE et al. s.n.; Sichuan, China | KC415528 | KC415471 | KC415358 | FJ788624 | FJ788561 | KC415414 |
|  | *S. paraplesia* C.K. Schneid. var. sub*integra* C. Wang & P.Y. Fu | Chen JH 782; Xizang, China; KUN | KC415529 | KC415472 | KC415359 | KC415308 | KC415571 | KC415415 |
|  | *S. pentandra* L. | Chen JH 135; Heilongjiang, China; KUN | KC415530 | KC415473 | KC415360 | FJ788625 | FJ788530 | \| KC415416 \| \| --- \| |
| sect. *Salicaster* Dumortier | *S. lucida* | Chen JH 206; New Hampshire, USA; KUN | KC415521 | KC415464 | KC415351 | FJ788626 | FJ788562 | \| KC415407 \| \| --- \| |
| sect. *Salix* | *S. alba* L. | Chen JH 563; Beijing, China; KUN | KC415492 | KC415435 | KC415323 | KC415284 | KC415547 | KC415379 |
|  | *S. babylonica* L. | Chen JH 107; Yunnan, China; KUN | KC415496 | KC415439 | KC415327 | FJ788622 | FJ788532 | KC415383 |
|  | *S. bangongensis* C. Wang & C.F. Fang | Chen JH 537; Xizang, China; KUN, CANM | KC415497 | KC415440 | KC415328 | KC415287 | KC415550 | KC415384 |
|  | *S. qinghaiensis* Y.L. Chou | Chen JH 470; Qinghai, China; KUN, CANM | KC415531 | KC415474 | KC415361 | KC415309 | KC415572 | KC415417 |
|  | *S. capitata* Y.L. Chou & A.K. Skvortsov | Wang DC 028; Heilongjiang, China; KUN, CANM | KC415499 | KC415442 | KC415330 | KC415288 | KC415551 | KC415386 |
|  | *S. chienii* W.C. Cheng | Wang DC 655; Zhejiang, China; KUN | KC415505 | KC415448 | KC415336 | KC415293 | KC415556 | KC415392 |
|  | *S. chikungensis* C.K. Schneid. | Wang DC 712; Henan, China; KUN, CANM | KC415506 | KC415449 | KC415337 | KC415294 | KC415557 | KC415393 |
|  | *S. heteromera* Hand.-Mazz. | Chen JH 517; Yunnan, China; KUN, CANM | KC415514 | KC415457 | KC415345 | KC415297 | KC415560 | KC415401 |
|  | *S. jessoensis* Seemen | HB 547; Hokkaido, Japan; KUN | KC415518 | KC415461 | KC415348 | KC415300 | KC415563 | KC415404 |
|  | *S. koreensis* Andersson | Wang DC 271; Heilongjiang, China; KUN, CANM | KC415519 | KC415462 | KC415349 | KC415301 | KC415564 | KC415405 |
|  | *S. matsudana* Koidz. | Chen JH 559; Hunan, China; KUN | KC415523 | KC415466 | KC415353 | KC415303 | KC415566 | KC415409 |
|  | *S. sericocarpa* Andersson | Li H et al. s.n.; Xizang, China; KUN, CANM | KC415536 | KC415479 | KC415366 | KC415311 | KC415574 | KC415422 |
|  | *S. sphaeronymphoides* Y.L. Chou | Chen JH 909; Xizang, China; KUN, CANM | KC415539 | KC415482 | KC415369 | KC415314 | KC415576 | KC415425 |
|  | *S. weixiensis* Y.L. Chou | Chen JH 926; Yunn?an, China; KUN, CANM | KC415545 | KC415488 | KC415375 | KC415319 | KC415581 | KC415431 |
| **subg. *Chamaetia*** A.K. Skvortsov |  |  |  |  |  |  |  |  |
| sect. *Floccosae* K.S. Hao ex C.F. Fang & A.K. Skvortsov | *S. floccosa* Burkill | Chen JH 063; Yunnan, China; KUN | KC415511 | KC415454 | KC415342 | FJ788597 | FJ788557 | KC415398 |
| sect. *Lindleyana* C.K. Schneid. | *S. gyirongensis* S.D. Zhao | Chen JH 155; Sichuan, China; KUN | KC415513 | KC415456 | KC415344 | FJ788605 | FJ788546 | KC415400 |
|  | *S. clathrata* Hand.-Mazz. | Chen JH 055; Yunnan, China; KUN | KC415508 | KC415451 | KC415339 | FJ788604 | FJ788536 | KC415395 |
| sect. *Retusae* A. Kerner | *S. nummularia* Andersson | Meng Y et al. s.n.; Jilin, China; KUN | KC415534 | KC415477 | KC415364 | FJ788611 | FJ788547 | KC415420 |
| sect. *Sclerophyllae* C.K. Schneid. | *S. sclerophylla* Andersson | Chen JH 152; Sichuan, China; KUN | KC415535 | KC415478 | KC415365 | FJ788616 | FJ788559 | KC415421 |
| **subg. *Vetrix*** A.K. Skvortsov |  |  |  |  |  |  |  |  |
| sect. *Cinerella* Ser. | *S. caucasica* Andersson | Sun H s.n.; Georgia; KUN | KC415501 | KC415444 | KC415332 | KC415289 | KC415552 | KC415388 |
| sect. *Incubaceae* Dumort. | *S. brachypoda* Komarov | Chen JH 0502; Jilin, Chin; KUN | KC415498 | KC415441 | KC415329 | FJ788607 | FJ788551 | KC415385 |
| sect. *Vetrix* Dumort. | *S. raddeana* Laksch. ex Nas. | Chen JH 142; Jilin, China; KUN | KC415532 | KC415475 | KC415362 | FJ788618 | FJ788550 | KC415418 |
| sect. *Vimen* Dumort. | *S. siuzevii* Seemen | Chen JH 131; Jilin, China; KUN | KC415537 | KC415480 | KC415367 | KC415312 | FJ788543 | KC415423 |

^a^ The subgeneric division of *Salix* follows Argus (2010), while the sectional division follows regional floras, such as *Flora of China* (Fang et al., 1999) and *Flora of North America* (Argus, 2010).

^b^ These materials were obtained from specimens already deposited in herbariums.

^c^ In the *Salix* system of Ohashi (2001), *Salix nipponica* Franchet & Savatier is a synonym of *Salix triandra* L.

^d^ Abbreviations: KUN, Herbarium of Kunming Institute of Botany, Chinese Academy of Sciences; A, Harvard University Herbaria; CANM, Herbarium of Canada Museum of Nature; s.n., *sine numero* (specimen unnumbered)
